# Offensive Transitions in High-Performance Football: Differences Between UEFA Euro 2008 and UEFA Euro 2016

**DOI:** 10.3389/fpsyg.2019.01230

**Published:** 2019-06-18

**Authors:** Rubén Maneiro, Claudio A. Casal, Isaac Álvarez, José Enrique Moral, Sergio López, Antonio Ardá, José Luís Losada

**Affiliations:** ^1^Faculty of Science of Education, Pontifical University of Salamanca, Salamanca, Spain; ^2^Department of Science of Physical Activity and Sport, Catholic University of Valencia “San Vicente Mártir”, Valencia, Spain; ^3^Department of Physical and Sports Education, University of A Coruña, A Coruña, Spain; ^4^Department of Methodology for Behavioral Sciences, University of Barcelona, Barcelona, Spain

**Keywords:** offensive transitions, football, high performance, mixed methods, observational methodology

## Abstract

Coaches, footballers and researchers agree that offensive transitions are one of the most important moments in football today. In a sport where defense over attack dominates, with low scores on the scoreboard, the importance of these actions from the offensive point of view becomes very important. Despite this, scientific literature is still very limited on this topic. Therefore, the objectives set out in the present investigation have been two: first, by means of a proportion analysis and the application of a chi-square test, it was intended to describe the possible differences between the offensive transitions made in the UEFA Euro 2008 and UEFA Euro 2016; then, through different multivariate analyzes based on logistic regression models, it was intended to know the possible differences among the proposed models. Using observational methodology as a methodological filter, 1,533 offensive transitions corresponding to the observation of the quarter final, semifinal, and final quarter of UEFA Euro 2008 and UEFA Euro 2016 have been analyzed. The results obtained have shown that offensive transitions between both championships have changed throughout both UEFA Euro, as well as some of the variables or behaviors associated with them (*p* < 0.05). The predictive models considered, although they have been developed from the same predictor variables, have also yielded different results for both championships, evidencing predictive differences among themselves. These results allow to corroborate that the offensive phase in high level football, specifically in what refers to moments of transition defense-attack, have evolved over these 8 years. At the applied level, the results of this research allow coaches to have current and contemporary information on these actions, potentially allowing them to improve their offensive performance during competition.

## Introduction

In football, the reality of competition forces teams to operate dynamically with alternatives when they have possession of the ball or the opponent has it. Attack and defend are a cyclical and dichotomous continuum. Contrary to what happens in other sports, teams can opt for a tactical provision based on the almost total renunciation of the ball. The use or not of the ball through possession of the same is not marked by any temporary limit regulation, teams have full freedom to start or finish possession when they deem appropriate (Castelano, 2008).

Team general tactics imply a constant interaction between attack and defense patterns (Barreira et al., 2014b; Araújo and Davids, 2016; Maneiro and Amatria, 2018). The complex nature of these interactions (Duarte et al., 2012), conditions the passage from one phase to another, so it requires a time of adaptation, which includes differentiated behaviors in the case of defending after attacking (defensive transition), or attacking after defending (offensive transition).

An offensive transition (or defense-attack transition) is considered all technical-tactical actions that a team makes since regaining possession of the ball in play and seek to take advantage of the rival’s collective reorganization (which is at that moment in defensive transition), to achieve an optimal progression situation of the ball and/or end, until it is organized offensively (organized attack) or the opponent is reorganized defensively (organized defense) (Casal et al., 2015).

In today’s football, the importance of the attack that starts with an offensive transition has experienced an increasing importance, according to several works (Mombaerts, 2000; Gréhaigne, 2001; Carling et al., 2005; Yiannakos and Armatas, 2006; Acar et al., 2009; Armatas and Yiannakos, 2010; Tenga et al., 2010b; Barreira et al., 2013; Leite, 2013; Plummer, 2013; Sarmento et al., 2014; Casal et al., 2015; Winter and Pfeiffer, 2015; Sgrò et al., 2016; Fernández-Navarro et al., 2018).

The purpose of offensive transitions varies according to the needs and will of the team that executes them. A direct and rapid offensive transition, with immediate goal search, is associated with two types of offensive end-of-play behaviors: counterattack and direct attack. On the other hand, an elaboration offensive transition, without immediate search of goal, and attack or defense not presenting organized patterns, is associated with progression behaviors toward the attack or as a means to reach various offensive subprinciples (Tenga et al., 2009, 2010c; Fernández-Navarro et al., 2018).

Offensive transition moments become unique actions due to the fluidity of the game’s dynamics. These are situations of role change, of an open nature, and to which we should add special spatial conditions (the game action takes place in wide spaces) and temporary ones (these are actions that are usually carried out at high speeds) (Lago et al., 2012). In addition, they are actions that emerge from a certain disorder, from role change because of the change in ball possession.

It is important to remember that the game is a continuous, cyclical and non-linear process, where attack, defense and transitions do not exist separately. Some phases condition and are conditioned by others. The defensive moment of the game begins before the loss of the ball, just as the offensive moment begins before the recovery. Therefore, a rational occupation of the strategic space is important, as well as knowing the rival’s tactical behavior. Whenever there is an offensive transition, there is an antagonistic response from the opposing team, in the form of a defensive transition (Vogelbein et al., 2014; Winter and Pfeiffer, 2015; Casal et al., 2016).

Works that have studied attack mechanisms in football confirm that attacks in transition (rapid attacks or counterattacks) have greater chances of success (goals scored, throws to goal or arrivals to the area) than other attack styles (Tenga et al., 2010a,b; Barreira et al., 2013; Sgrò et al., 2017; Fernández-Navarro et al., 2018).

Analyzing in detail behaviors that modulate or condition the effectiveness of these actions, preceding works have highlighted a series of variables that teams must take into account.

The beginning zone of the offensive transition has been analyzed in different studies. The vast majority of literature has agreed that offensive success effectiveness increases the closer to the rival goal the transition is achieved (Tenga et al., 2010a,b; Lago et al., 2012) although with moderate differences depending of the starting sector (James et al., 2002; Barreira et al., 2014b; Casal et al., 2016). Probably this lack of consensus is provoked by different proposals of field division.

With regard to the progression strategy to rival goal or conservation immediately after ball recovery, most of the available data confirm that rapid and direct progression is the most effective behavior, both when producing area arrivals as goals attainment (Tenga et al., 2010a,b; Zurloni et al., 2014; Casal et al., 2015). Although works that disagree with these results should also be taken into account (Tenga et al., 2010c; Sgrò et al., 2016).

Regarding the sequence of passes used in the offensive transition, different results are also found among the scientific community. In this sense, a large majority of publications emphasize that the use of a small number of passes constitutes the most effective offensive procedure (≤4 passes) (Mombaerts, 2000; Acar et al., 2009; Lago et al., 2012), although there are works that reject these results (Tenga et al., 2010c; Barreira et al., 2014b).

Finally, with regard to the transition duration, the available data allows us to speak of a general consensus among different authors. Thus, practically all studies conclude that they must be actions developed at high speed to be successful (Wallace and Norton, 2014), with a temporal margin that varies between 1″ and 5″ (Gréhaigne, 2001; Hughes and Churchill, 2005; Acar et al., 2009) and ≤15″ (Garganta et al., 1997; Carling et al., 2005).

All the data and evidence presented have highlighted the importance of offensive transitions during matches. At the methodological level, many of the works consulted are of a quantitative nature (motion analysis), based on competition description through element or behavior frequency. In this work, in addition to a quantitative analysis, a complementary qualitative analysis will be carried out, thus providing greater uniqueness and a more objective and holistic view to the study of the football reality and transitions in particular. For this, the ideal option is systematic observation, thus ensuring a balance between the robustness of quantitative data, and the flexibility provided by qualitative data, in order to make a more objective approach of the observed reality. For this, the present study starts from a *mixed methods* perspective (Johnson et al., 2007; Creswell, 2011; Creswell and Plano-Clark, 2011; Freshwater, 2012; Anguera et al., 2018b).

The integration of quantitative and qualitative data from the mixed methods perspective will allow proposing a holistic and integral model, allowing a more objective approach of the observed reality. Systematic observation, both direct and indirect, provides qualitative information on the registry, focused, respectively, on transition quality and previous documents (Gorard and Makopoulou, 2012; Anguera et al., 2017, 2018a), which will be followed by a second quantitative stage (data quality control and data analysis), to recover the initial objective by discussing results. In this way, a new methodological alternative to the study of football and its different manifestations is opened, proposing solutions to the aforementioned complex reality (Duarte et al., 2012).

The observational methodology application will achieve the objectives set out in the present work, which are: on the one hand, to know the differences in terms of regularity and usual execution practices in offensive transitions executed during the European Championship of Nations Euro 2008 and UEFA Euro 2016; and, on the other hand, by performing different multivariate analysis, design an execution model of the offensive transitions with greater probabilities of success, for both championships, and identify the differences between both models.

## Materials and Methods

### Design

Among the possible designs that can be presented by the observational methodology, a nomothetic, intersessional monitoring and multidimensional design was applied (Anguera, 1979). Nomothetic because a plurality of units are studied, intersessional over time and multidimensional because we analyzed the multiple dimensions that constituted the *ad hoc* observation instrument used.

The systematic observation carried out has been non-participant and active, using an observational sampling “all occurrence”.

### Participants

In this study, the analysis unit is the defense-attack transitions in top-level football. The observation sample was a convenience sample (Anguera et al., 2011). We analyzed 1,533 events corresponding to the observation of 14 matches, during the Quarter-finals, semi-finals and finals of UEFA Euro 2008 and UEFA Euro 2016. These matches are played in the direct elimination mode, which causes both teams to need offensive attack procedures to achieve a positive result.

### Observation Instrument

The observation instrument proposed by Casal (2011) was used. In it, the inclusion and exclusion criteria can be consulted. This observation instrument is made up of a combination of field formats and category systems, where the dimensions of the instrument’s categories and the inclusion and exclusion criteria can be consulted.

Data was collected and coded using the LINCE software (v 1.2.1, Gabin et al., 2012). The IBM SPSS Statistics 25 program for descriptive and bivariate analysis and the R program for multivariate analysis were used as analysis tools. Finally, the STATGRAPHICS Centurion program, v16, was used to analyze proportions.

### Procedure

The meetings were recorded from images broadcast on television.

According to the Belmont Report (National Commission for the Protection of Human Subjects of Biomedical, and Behavioral Research, 1978) the use of public images for research purpose does not require informed consent or approval of an ethical committee.

There were four observers selected for data collection, four of them being doctors in Sports Science. Three are national soccer coaches, and also with more than 5 years of experience in the use and application of observational methodology. Prior to the coding process, observers were trained during eight training sessions (Losada and Manolov, 2015; Manolov and Losada, 2017), applying the consensual agreement criterion among observers and were provided with a specifically designed observation protocol.

### Data Quality Control

The quality control of the data was carried out using the IBM SPSS Statistics 25. To try to ensure data reliability, all matches were registered and analyzed by four observers, three of them national soccer coaches with years of experience in the field of training, teaching, and research in football through observational methodology. In addition, the following training process was carried out. First, eight observing sessions were conducted on teaching the observers following the Losada and Manolov (2015) criteria and applying the criterion of consensual agreement (Anguera, 1990) among observers, so that recording was only done when agreement was produced. To ensure inter-reliability consistency of the data (Berk, 1979), the Kappa coefficient was calculated for each criterion (Table 1), it revealed a strong agreement between observers, which means high reliability, taking Fleiss et al. (2003) as a reference.

**Table 1:** The interobserver agreement analysis for each criterion.

Criteria	Ob_1_–Ob_2_	Ob_1_–Ob_3_	Ob_1_–Ob_4_	Ob_2_–Ob_3_	Ob_2_–Ob_4_	Ob_3_–Ob_4_
Start of possession	0,82	0,83	0,86	0,82	0,87	0,91
Interaction context	0,74	0,74	0,81	1	0,76	0,75
Defensive organization	0,81	0.85	0,83	0,78	0,8	0,8
Time	1	0,96	1	0,84	1	0,81
Intention	0,8	0,82	0,72	0,91	0,87	0,82
Number of Intervening	0,82	0,72	1	0,86	0,72	1
Number of passes	1	0,80	0,80	0,92	1	0,86
Final interaction context	0,76	0,81	0.92	0,81	0,83	0,81
Match status	0,9	1	1	1	1	0,88
Success	1	1	0,88	1	0,9	1
*K*_total_	0,86	0,85	0,88	0,87	0,81	0,86

### Data Analysis

As regards data analysis, and in accordance with the objectives set, three types of analysis were defined: by means of a proportion analysis and the application of a chi-square test, the aim was to describe differences on the championship level, and the modulating variables level, in the execution and habitual practices of the offensive transitions between both championships. Then, three types of analysis were carried out: a logistic regression to know the variables that may be modulating the effectiveness achieved; the Mcfadden test was applied to check the model’s goodness of fit. Finally, an ANOVA analysis was implemented to analyze the variance and deviation table. The aim is to know the differences between successful models for UEFA Euro 2008 and UEFA Euro 2016.

## Results

**FIGURE 1:**
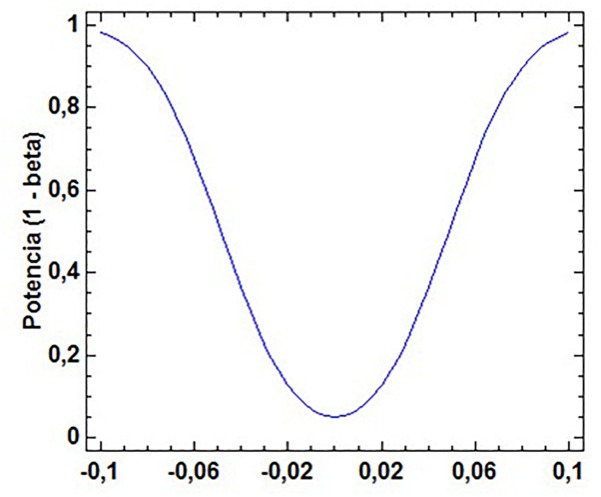
Proportion analysis for the UEFA Euro 2008 and UEFA Euto 2016 samples. Power curve (alpha = 0.05, average ratio = 0.381012).

First, a proportion comparison (Figure 1) has been carried out using the binomial test. Data presented in Figure 1 shows statistically significant differences between the UEFA Euro 2008 and UEFA Euro 2016 championships (sample proportions = 0.347 and 0.413, sample size = 743 and 790).

Statistics *z* calculated = -2.65932; *p* = 0.007.

**FIGURE 2:**
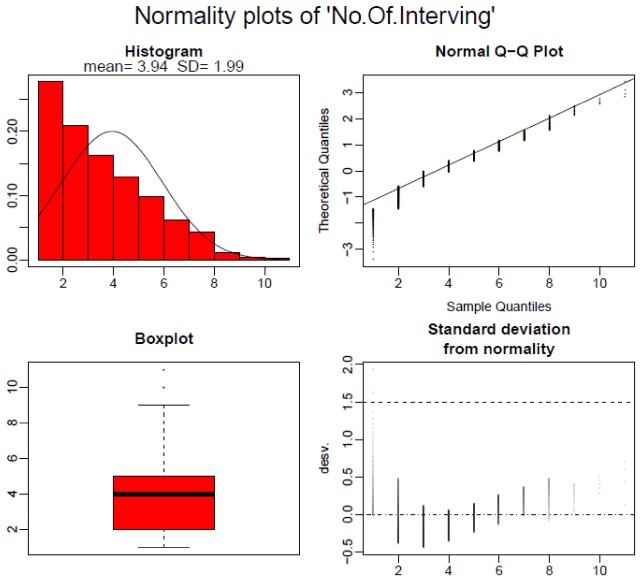
Distribution for the quantitative variable Number of Intervening. Shapiro–Wilks *p*-value: <001.

**FIGURE 3:**
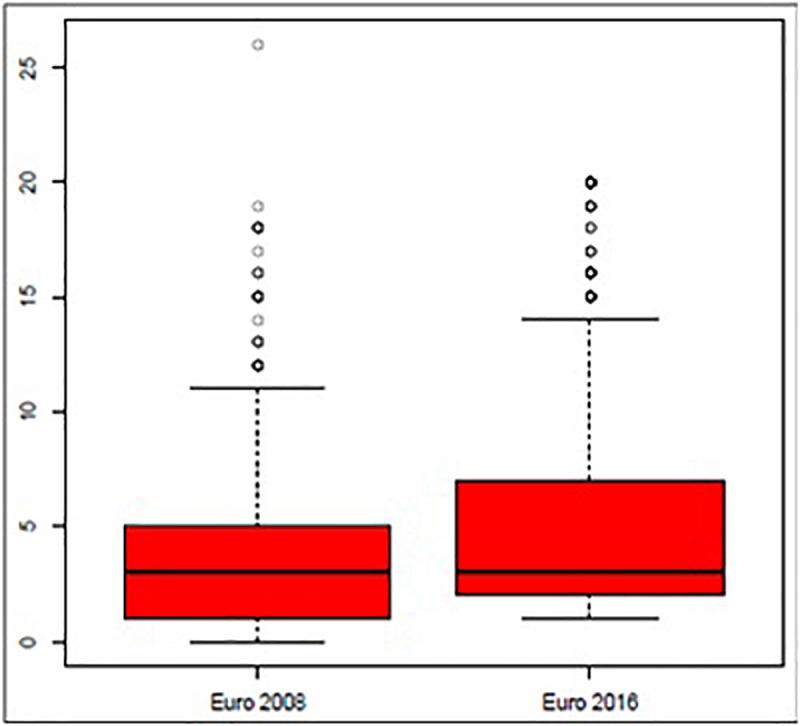
Boxplot of Number of Intervening by competition.

On the other hand, data presented in Table 2 shows statistically significant differences between variables considered for each championship. Specifically, there are eight variables that present significant differences between both championships: “Start of possession” (*p* < 0.001), “Interaction Context” (*p* < 0.001), “Defensive Organization” (*p* < 0.001), “Intention” (*p* < 0.001), “Number of passes” (*p* < 0.001), “Final Interaction Context” (*p* < 0.001), “Match status” (*p* < 0.001) and “Success” (*p* = 0.008). The quantitative variable “No. of Intervening” does not follow a normal distribution (Figure 2, 3).

**Table 2:** Summary descriptives table by groups of “competition”.

	Euro 2008	Euro 2016	*p*. overall
	*N* = 743	*N* = 790	
**Start of possession:**			<0.001
Defensive	230 (31.0%)	215 (27.2%)	
MD	332 (44.7%)	195 (24.7%)	
Central	120 (16.2%)	235 (29.7%)	
MO	56 (7.54%)	133 (16.8%)	
Ofensiv	5 (0.67%)	12 (1.52%)	
**Interaction Context**			<0.001
PA	132 (17.8%)	113 (14.3%)	
RA	262 (35.3%)	291 (36.8%)	
RM	53 (7.13%)	14 (1.77%)	
MR	7 (0.94%)	36 (4.56%)	
MM	250 (33.6%)	249 (31.5%)	
MA	12 (1.62%)	33 (4.18%)	
AR	21 (2.83%)	41 (5.19%)	
AM	5 (0.67%)	13 (1.65%)	
A0	1 (0.13%)	0 (0.00%)	
**Defensive organization**			<0.001
Organized	604 (81.3%)	451 (57.1%)	
Circums	139 (18.7%)	339 (42.9%)	
**Time**			0.491
0–30	248 (33.4%)	271 (34.3%)	
31–60	219 (29.5%)	223 (28.2%)	
61–90	202 (27.2%)	232 (29.4%)	
91–120	74 (9.96%)	64 (8.10%)	
**Intention:**			<0.001
Progress	370 (49.8%)	613 (77.6%)	
Conserve	373 (50.2%)	177 (22.4%)	
Number of Intervening	4.00 [2.00; 5.00]	4.00 [2.00; 5.00]	0.907
Number of passes	3.00 [1.00; 5.00]	3.00 [2.00; 7.00]	<0.001
**Final Interaction context**			<0.001
PAF	21 (2.83%)	17 (2.15%)	
RAF	56 (7.54%)	13 (1.65%)	
RMF	10 (1.35%)	23 (2.91%)	
MRF	24 (3.23%)	26 (3.29%)	
MMF	280 (37.7%)	198 (25.1%)	
MAF	4 (0.54%)	7 (0.89%)	
ARF	312 (42.0%)	451 (57.1%)	
AMF	12 (1.62%)	22 (2.78%)	
A0F	24 (3.23%)	33 (4.18%)	
**Match status**			<0.001
Winning	101 (13.6%)	195 (24.7%)	
Drawing	538 (72.4%)	404 (51.1%)	
Losing	104 (14.0%)	191 (24.2%)	
**Success**			0.008
No success	485 (65.3%)	463 (58.6%)	
Success	258 (34.7%)	327 (41.4%)	

Finally, an analysis was applied based on a logistic regression model (Tables 3, 4) configured by the same predictor and explained variables, for both UEFA Euro 2008 and UEFA Euro 2016 championships, in order to be able to compare which variables are significant in achieving success, and knowing whether or not they are the same, in both competitions, based on their *Deviances*.

**Table 3:** Analysis of deviance table.

	Df	Deviance Resid.	Df Resid	Dev	Rao	Pr (>Chi)
NULL			742	959.54		
Defensive organization	1	1.267	741	958.28	1.2835	0.2572498
Final interaction context	8	36.979	733	921.30	30.7907	0.0001531^***^
Intention	1	1.413	732	919.8	1.4155	0.2341398
Interaction context	8	13.117	724	906.77	12.6617	0.1240335
Match status	2	0.378	722	906.39	0.3759	0.8286680
Number of passes	1	0.772	721	905.62	0.7830	0.3762162
Start of possession	4	2.626	717	902.99	2.6298	0.6215546

*Model, binomial, link logit, and response success Significance codes: 0 ‘^∗∗∗^’ 0.001 ‘’ 1.*

**Table 4:** Analysis of deviance table.

	Df	Deviance Resid.	Df Resid.	Dev	Rao	Pr (>Chi)
NULL			742	1019.33		
Defensive organization	1	35.105	741	984.22	34.970	3.349e-09^∗∗∗^
Final interaction context	8	218.276	733	765.95	177.748	<2.2e-16^∗∗∗^
Intention	1	0.242	732	765.71	0.242	0.62296
Interaction context	7	16.688	725	749.02	15.478	0.03033^*^
Match status	2	0.818	723	748.20	0.817	0.66472
Number of passes	1	3.813	722	744.39	3.795	0.05139.
Start of possession	4	12.416	718	731.97	11.955	0.01769^*^

*Model, binomial, link logit, and response success Significance codes: 0 ‘^∗∗∗^’ 0.001 ‘^∗^’ 0.05 ‘.’ 0.1 ‘’ 1.*

Specifically, for the UEFA Euro 2008, the proposed model is:

Success = μ + β_1_ DefensiveOrganization + β_2_ FinalInteractionContext + β_3_ Intention + β_4_ InteractionContext + β_5_ MatchStatus + β_6_ NumberOfPasses + β_7_ StartOfPossession

The adjustment of the model is checked with the McFadden test with a value of 0.0589. The accuracy in the predictive capacity of the model is 0.918 (Accuracy).

Next, an Anova analysis was executed in the model to analyze the deviation table. By specifying a single model, a sequential analysis of the deviation table is made to fit. That is, the reductions in the residual deviation that is added to each model term, in addition to the residual deviations themselves.

The wider the difference between the zero deviation and the residual deviation, the better. Analysis of the table shows the descent of the deviation when adding each variable. The addition of “Final Interaction Context” significantly reduces the residual deviation. A large *p*-value indicates that the model without the variable explains more or less the same amount of variation. Ultimately, the optimum is a significant drop in deviation. Finally, the Rao efficient scoring test was applied, which has an asymptotic chi-square distribution to detect the most influential factors in success.

On the other hand, the proposed model for the UEFA Euro 2016 is:

Success = μ + β_1_ DefensiveOrganization + β_2_ FinalInteractionContext + β_3_ Intention + β_4_ InteractionContext + β_5_ MatchStatus + β_6_ NumberOfPasses + β_7_ StartOfPossession

The adjustment of the model is checked with the McFadden test with a value of 0.2819095. The accuracy in the predictive capacity of the model is 0.5128 (Accuracy).

An Anova analysis is performed in the model to analyze the deviation table. By specifying a single model, a sequential analysis of the deviation table is made to fit. That is, the reductions in the residual deviation that is added to each term of the formula, in addition to the residual deviations themselves.

The wider the difference between the zero deviation and the residual deviation, the better. Analysis of the table shows the descent of the deviation when adding each variable. The addition of “Defensive Organization,” “Final Interaction Context,” “Interaction Context,” “Number Of Passes,” and “Start Of Possession” significantly reduces the residual deviation. Finally, the Rao efficient scoring test was applied, which has an asymptotic chi-square distribution to detect the most influential factors in success.

In summary, variables that provide information to the explained variable “success,” in the case of Eurocopa 2008, is the predictive variable “Final Interaction Context”. The inclusion of other variables does not provide any variation in the model. In the case of Euro 2016, the variables “Defensive Organization,” “Final Interaction Context,” “Interaction Context,” “Number Of Passes,” and “Start Of Possession,” decrease the residual *deviance* and therefore are important in the model. Depending on the competition, all of them participate significantly in success achievement in the game.

## Discussion

The present work was proposed with the objective of identifying and describing possible differences in the execution of defense-attack transitions in one of the most important championship of nations: the UEFA Euro. For this, the editions of 2008 and 2016 have been analyzed. By performing different statistical analysis (accompanied by a proportion analysis, a chi-square contrast and various logistic regression analysis), and in view of the available data, it can be verified that these actions do present different behavior patterns between both championships.

In the first place, as regards the first of the stated objectives, it can be said that significant differences in a championship level between UEFA Euro 2008 and UEFA Euro 2016 are found. In particular, during the last championship there has been an increase in 6.32% of the number of offensive transitions (*p* = 0.007) compared to the 2008 championship. These results allow us to think that attack game dynamics have evolved toward open nature patterns, with the development of the game in wider spaces and with shorter offensive actions. This occurs to the detriment of more elaborate attack mechanisms, where high defensive density and reduced time in decision-making hinders the creation of favorable superiority contexts (Wallace and Norton, 2014; Barreira et al., 2015; Casal et al., 2017). As a consequence, it is plausible to affirm that new teams take advantage of the possible moments of uncertainty and stress caused by the role change during the game. Furthermore, this change in attack mechanisms has probably also emerged answering to new scenarios in the environmental conditions of the game, such as the partial result of the game, competition type or the opposing team quality (Lago, 2009).

Finally, available results allow to qualify works where it is concluded that soccer has barely changed in the last decades (Castellano et al., 2008). This work corroborates previous works in which the evidence of football evolution has been contrasted (Wallace and Norton, 2014; Barreira et al., 2014a, 2015).

About the second objective, to know differences between the efficiency degree achieved and the different variables considered in both championships, it has been detected that teams have experienced a marked evolution regarding the beginning zone of the transition, passing from the mid-defensive zone to the central area of the field. In addition, there has also been a noticeable increase in the recoveries that occur in the medium-offensive zone compared to the 2008 Euro edition. This circumstance may be due to a better management of the technical, tactical and physical player resources, since according to different works (Tenga et al., 2010a,b; Lago et al., 2012), the optimal zone of ball recovery is in the offensive midfield, especially in regions near the rival goal (Barreira et al., 2014a).

This fact causes less physical wear and less demand for complex tactical benefits (attack construction begins in areas close to the target). Ball recovery in this zone would allow a greater use of the game phase weaknesses in which the opponent is situated (attack construction and ball possession), that when being in attack deployment, this propitiates the appearance of larger spaces between the different lines (inter-lines), as well as between players of the same line (intra-line), a circumstance that the defending team can take advantage of at the moment of role change (move to attack after defending) to advance or finalize the action. In addition, the ball recovery in areas close to the rival goal means, in most cases, that the attacking team will only have to overcome the rival defensive line or, at most, this one plus the middle line.

With regard to the “Interaction Context” in which the defense-attack transition begins, several authors have highlighted the importance of motor interaction analysis in football (Castellano and Hernández-Mendo, 2003; Garganta, 2009; Sarmento et al., 2018). In view of the available data, it is possible to verify that teams have significantly modified their spatial configuration of interaction, resulting in greater recoveries in the most offensive contexts considered (AM, AR, MA, and MR). These data corroborate the work of Casal et al. (2015), which finds worse data in success terms in the PA category; Almeida et al. (2014), who observe that successful teams recover the ball in areas close to the rival goal: and Castellano and Hernández-Mendo (2003), who associate the MR variable as of great offensive value. Finally, the MM and RA categories continue to appear as the most regular, that is, losses usually occur in the middle and advanced line of the observed team. The frequency of the MM category during transitions allows us to think that it is a transition category, where the team attacks flow with a certain offensive character.

The third variable that has shown significant differences has been the type of “Defensive Organization”. Available data of both championships allow to verify a robust evolution in defensive mechanisms. Specifically, the increase of 24.2% of circumstantial defense after role change in ball possession with respect to the 2008 edition allows to speak of two antagonistic aspects: on the one hand, a possible defensive flexibilization is verified by part of the teams, which accept the inherent risks of circumstantial defense (greater defensive disorder and incorrect management of strategic spaces) in favor of potentially greater offensive features (larger spaces and more players in a position to carry out an attack); on the other hand, it identifies possible defensive weaknesses in the teams when they pass from attack to defense. These weaknesses are far from the studies of Casal et al. (2016), where the importance of the defensive transition in the moments following the loss of the ball is highlighted; and from Winter and Pfeiffer (2015), where they affirm that part of the winning teams’ success is due to high performance in these transitions. Transition effectiveness is related to team organization before them.

On the other hand, significant results were found regarding the “Tactical Intention” of the team immediately after ball recovery. Although in the 2008 edition teams opted for a balanced disposition between progressing toward a rival goal or keeping the ball at the beginning of the offensive transition, 8 years later teams have a clear desire to progress toward offensive areas. These data could be directly related to the variable “Defensive Organization,” it is possible that a defensive behavior causes the appearance of an offensive and antagonistic behavior in the rival team, and vice versa. On the other hand, the works of Tenga et al. (2010b) and Lago et al. (2012), state that counterattacks are the ideal attack against disorganized defenses. On the other hand, it should be noted that the importance of this variable is still under debate in scientific literature (Tenga et al., 2010c; Sgrò et al., 2016; Sgrò et al., 2017).

Regarding the “Number of Passes” variable, results show significant differences. In particular, it is possible to highlight an increase in the variance of these actions in the Euro 2016 edition. This corroborates previous works such as those of Barreira et al. (2014b), which currently confirm a new tactical alternative in teams, based on greater collective behavior in offensive transitions; and Tenga et al. (2010c), who report higher efficiency rates with long possessions (>5 passes).

The “Final Interaction Context,” the spatial configuration of both teams at the moment in which the observed team finishes its offensive sequence, presents significant differences (<0.001). Lower finalization rates have been found in the MMF context, and instead higher rates (15% more) of offensive transitions ending in the ARF context are collected, as well as better results for the AMF and A0F contexts. Again, this behavior reinforces the evidence presented: in these 8 years, teams have greater offensive will.

In regard to the “Partial Result of the Match,” variable regularly collected in scientific literature, it is possible to refer significant changes (<0.001). Despite the fact that a large part of the offensive transitions are executed with a draw (51.1%), a strong evolution is observed in comparison to the 2008 championship. In particular, one of every two offensive transitions occurs with an imbalance in the scoreboard. A possible explanation could be found in that this imbalance occurs in very early phases of the game, which rival teams fail to neutralize in successive instants, thus promoting the existence of more effective time with an unbalanced score. Another possible explanation could lie in the fact that teams in the UEFA Euro 2016 have higher rates of circumstantial defense (<0.001), a situation that could cause attack levels to be above defense levels. Finally, the possible team heterogeneity in terms of quality, which promotes unstable markers on a regular basis as a last possible explanation.

Finally, “Success” rates at UEFA Euro 2016 are significantly higher than at UEFA Euro 2008. 41.4% of offensive transitions have been successful, in comparison to 34.7% at UEFA Euro 2008. It is worth remembering that this paper collects performance indicators collected in Casal et al. (2015) as success.

In short, empirical data reveals the tactical alternative success of the teams that have opted for defense-attack transitions with a marked finalizing character, with an immediate search for auction opportunities, and away from speculative or containment behavior. Teams have opted for the alternative of initiating transitions in more advanced areas of the field, a moderate variance in the number of passes and finalizing actions in more offensive contexts.

Finally, the multivariate analysis carried out has allowed to verify the alternative of the explanatory variables that intervene in the presented models (Tables 3, 4). The test allowed us to measure the extent to which the accuracy of the complete model improves compared to the reduced model. In this case, for the UEFA Euro 2008, the predictive capacity of the model is very good (0.918), although the only variable that provides information is the “Final Interaction Context,” having a low adjustment level. These results highlight the importance of deciding on which line of the observed team the offensive transition ends and in which defensive line of the rival team it is important to establish this interaction. Some possible explanations for the importance of finishing offensive transitions against the middle or delayed line of the rival team is that the opponent has fewer players in position to defend; on the other hand, if an offensive transition finalizes in this context, it is probably because the ball has been stolen from the midline or delayed line and the defending team is probably disorganized. Finally, it is also congruent to think, in view of the data and the strength of the variable explained, that this finalization should occur in offensive areas, close to the rival goal, as the best means to achieve success. Although it must be taken into account that the model is more efficient taking all variables in their entirety (Table 3).

In contrast, for UEFA Euro 2016, although worse values are found in predictive terms, the model has a more robust overall adjustment. In applied terms, it is possible to explain the success of these actions taking into account five variables (“Defensive Organization,” “Final Interaction Context,” “Interaction Context,” “Number Of Passes,” and “Start Of Possession”). This way, the model explains 51% of the offensive transitions in the 2016 championship, with a higher adjustment than the 2008 championship model. In practical terms, it is plausible to think that the results emphasize the importance of the team’s tactical construction, based on a refined space management on where to recover the ball and interact with the opponent, the number of precise passes and the need to know the defensive behavior of the opponent. Also, the inclusion of the variable “Final Interaction Context” in the second model reinforces the suitability of where to end the offensive transition, and against which line of the rival team. It is likely that this finalization, as in the 2008 championship, should occur in offensive areas (AR, front line of the observed team against the opponent’s delayed line).

## Conclusion

The main conclusions that can be drawn from this work can be summarized in: (1) Football is not a sport that experiences regular and stable behavior, but behaves like a living organism, which changes and evolves over time. (2) The success of the offensive transitions in the 2016 edition is greater than in the 2008 edition. (3) Offensive transitions executed in the UEFA Euro 2016 present significantly more offensive behavior than in the 2008 edition. (4) The multivariate model presented for the 2008 edition better predicts offensive transitions in their entirety, but their adjustment is moderate; on the other hand, the model presented for the 2016 competition has worse predictive capacity, but greater adjustment in its entirety.

## Limitations

First, it is important to note that the goodness of fit of the explanatory models presented is moderate. Another of the present limitations has to do with the generalization degree of the results or external validity of the same, given that actions corresponding to only one specific competition were selected as the unit of analysis: the UEFA Euro.

## Future Lines of Research

The future lines of research that can be derived from this study include the incorporation of new variables such as possession duration, individual technical behavior of different players and the proposal of a playing field zoning to prioritize optimal spaces to execute offensive transitions. Finally, it would be interesting to perform comparative analysis with domestic league competitions. Undoubtedly, the incorporation of these variables should help reduce the error component in the different models.

## Author Contributions

RM and IÁ collected the data, reviewed the literature, and wrote the manuscript. CC, SL, and JM reviewed the literature and supervised the work critically. JL analyzed the data and performed statistical analyzes. AA performed the method.

## Conflict of Interest Statement

The authors declare that the research was conducted in the absence of any commercial or financial relationships that could be construed as a potential conflict of interest.
